# Accurate prediction of protein enzymatic class by N-to-1 Neural Networks

**DOI:** 10.1186/1471-2105-14-S1-S11

**Published:** 2013-01-14

**Authors:** Viola Volpato, Alessandro Adelfio, Gianluca Pollastri

**Affiliations:** 1 School of Computer Science and Informatics, University College Dublin, Ireland; 2 Complex and Adaptive Systems Laboratory, University College Dublin, Ireland

## Abstract

We present a novel ab initio predictor of protein enzymatic class. The predictor can classify proteins, solely based on their sequences, into one of six classes extracted from the enzyme commission (EC) classification scheme and is trained on a large, curated database of over 6,000 non-redundant proteins which we have assembled in this work. The predictor is powered by an ensemble of N-to-1 Neural Network, a novel architecture which we have recently developed. N-to-1 Neural Networks operate on the full sequence and not on predefined features. All motifs of a predefined length (31 residues in this work) are considered and are compressed by an N-to-1 Neural Network into a feature vector which is automatically determined during training. We test our predictor in 10-fold cross-validation and obtain state of the art results, with a 96% correct classification and 86% generalized correlation. All six classes are predicted with a specificity of at least 80% and false positive rates never exceeding 7%. We are currently investigating enhanced input encoding schemes which include structural information, and are analyzing trained networks to mine motifs that are most informative for the prediction, hence, likely, functionally relevant.

## Background

Genome sequencing projects and high-throughput experimental procedures have produced a rapid growth in protein databases but only a small fraction of known sequences have been determined to have a function by experimental means. Determining or accurately predicting protein functions and enhancing the annotation of sequence databases is thus of paramount importance, in order to expand our knowledge of the mechanisms of life and to develop new drugs [1]. In spite of substantial interest by the research community in the prediction of protein functions, this, to date, remains a difficult problem for a number of reasons, partly because function itself is to an extent ill-defined, partly because we still lack a complete understanding of the complex relationship between sequences, structures and functions. There are numerous examples of divergent or convergent evolutionary events which, respectively, lead to closely related proteins with substantially different functions and to proteins with very different folding patterns that share the same function [2,3]. The TIM-barrel fold, for instance, is known to be shared by many enzymes of known structure and if in some cases it might have been adopted by convergent evolution because of its stable and useful characteristics, it also appears in homologous enzymes which have diverged to assume different functions [4]. By converse, the catalytic triad Ser-His-Asp appears by convergent evolution in many non-homologous proteins such as two proteinases (chymotrypsin and subtilisin) which have different folds but share similar catalytic functions [1].

However, a large and growing amount of annotated biological data is available to try to shed light on these issues and construct methods for automated function prediction. If some predictive methods rely on amino acid sequence analysis only, others take advantage of physio-chemical and structural properties or phylogenetic information and protein interactions while many others rely a combination of multiple data types. Traditionally, predicting protein function from the three-dimensional structure has been the most successful method but, since protein structures are known for less than 1% of known protein sequences, most proteins of newly sequenced genomes have to be characterized by their amino-acid sequences alone [1].

Techniques to predict protein function from sequence can be categorized into three main classes: sequence homology-based approaches, subsequence-based approaches and feature-based/ab-initio approaches. In homology-based approaches an annotation transfer can be proposed with confidence for the query protein [5] when the query shows high sequence similarity to a family of proteins of known function. In order for these methods to be successful the function of the homologous family of proteins must have been determined experimentally and, especially, the level of sequence similarity should be typically higher than that required for transferring structural annotation [6,7]. How much higher also depends on the type of function to be transferred, though a minimal sequence identity of 40% has been proposed [8-10]. This limits the applicability of this class of methods to a small fraction of all known protein sequences. When homology-based methods are not applicable, subsequence-based approaches, relying on multiple sequence alignments, can identify conserved residues, sequence signature patterns and sequence motifs crucial to determine the functional role of proteins [11]. Especially frequency profile methods, weighting residue positions according to the variability of their contents in a group of homologous proteins, may be sensitive detectors of distant homology [12]. In feature-based approaches, instead, information about function is assumed to be predictable via a range of features of proteins, including secondary structure, post-translational modifications and general properties of the amino-acid composition [1]. These features are differently combined to select only those that are mostly informative, non redundant and minimally noisy and usually processed by Machine Learning techniques such as Support Vector Machines and Neural Networks. Among feature-based approaches, one of the most cited works is [13] where Neural Networks are used to extract these features from protein sequences and the results are correlated with functional and enzymatic classes. Neural Networks, indeed, are one of the best suited computational algorithms for the classification of biological data thanks to their capability to identify patterns and correlations, their being error-and noise-tolerant and able to deal with large volumes of data [14].

Recently we have developed SCLpred [15], a predictor of protein Subcellular Localization (a simple definition of protein function) powered by a novel Neural Network architecture we have introduced and called N-to-1 NN. The main appeal of the N-to-1 NN model is that it is capable of automatically extracting small sets of highly informative features from the subsequences of a protein, thus combining advantages of feature-based and subsequence based methods. In our tests SCLpred matches or outperforms the state of the art in subcellular localization prediction. In another independent study N-to-1 NN were found to improve the state of the art in detecting transmembrane *β*-barrel chains [16].

Here we present a predictive method, based on N-to-1 NN, which is able to classify animal proteins into six enzymatic classes derived from the enzyme commission (EC) classification scheme. Because the selection of an appropriate input coding method is one of the most significant factors determining the performance of the prediction [14], we represent each sequence position by the residue frequency derived from multiple sequence alignments instead of using single protein sequences, thus exploiting the additional information contained in patterns of residue substitutions that reflect the family's features, such as three-dimensional fold, development of protein complexes and participation in the same chemical pathways [17]. The performances we obtain are substantially higher than those reported in previous studies.

## Methods

### Dataset

The enzyme dataset used to train and test our method is created using the ENZYME database available at http://www.expasy.org/enzyme/ (released on 28/06/2011) which provides UniprotKB/SwissProt entries. Enzyme sequences are selected for only metazoa taxonomy group and, according to EC numbers, classified into six main classes describing the reaction they catalyze: oxidoreductase (EC.1), transferase (EC.2), hydrolase (EC.3), lyase (EC.4), isomerase (EC.5) and ligase (EC.6). In order to obtain high quality data, we exlude enzymes with the same EC numbers and with less than 50 amino acids; using an all-against-all BLAST search (with *e *= 10^−3^) to reduce redundancy, we eliminate enzymes with more than 30% sequence identity to any other in the same functional class in a first step and to any other in the entire dataset in a second step. The enzymatic sequence dataset consists finally of 6081 entries. We should stress that this is, to the best of our knowledge, the largest high-quality, curated, non-redundant dataset representing this set of enzymes, and we have generated it specifically for this work. The dataset is available upon request from the authors. Table 1 shows the number of sequences per class obtained after the preprocessing phase.

**Table 1:** Number of sequences per class in the dataset.

Metazoa dataset	
Oxidoreductase	954
Transferase	2110
Hydrolase	2226
Lyase	208
Isomerase	136
Ligase	445

Total	6081

#### Input coding

In order to add evolutionary information to our dataset, each sequence position is represented by the amino acid-residue frequency derived from multiple sequence alignments extracted from uniref90 [18] from February 2010 containing 6,464,895 sequences. The alignments are generated by three runs of PSI-BLAST with parameters *b *= 3000 (maximum number of hits) and *e *= 10^−3 ^(expectation of a random hit) as in [15]. The input presented to the networks is the frequency of each of the 20 non-gap symbols plus an extra input for the total frequency of gaps in each column of the multiple sequence alignment (MSA) for a first version of the dataset in which we provide only evolutionary information and which we call MSA-dataset. In a second version, called MSA+SS-dataset, 3 additional inputs are considered for each residue to include information on the secondary structures which the given residue is predicted to belong to by the Porter server [19-22].

### Predictive architecture

**Figure 1:**
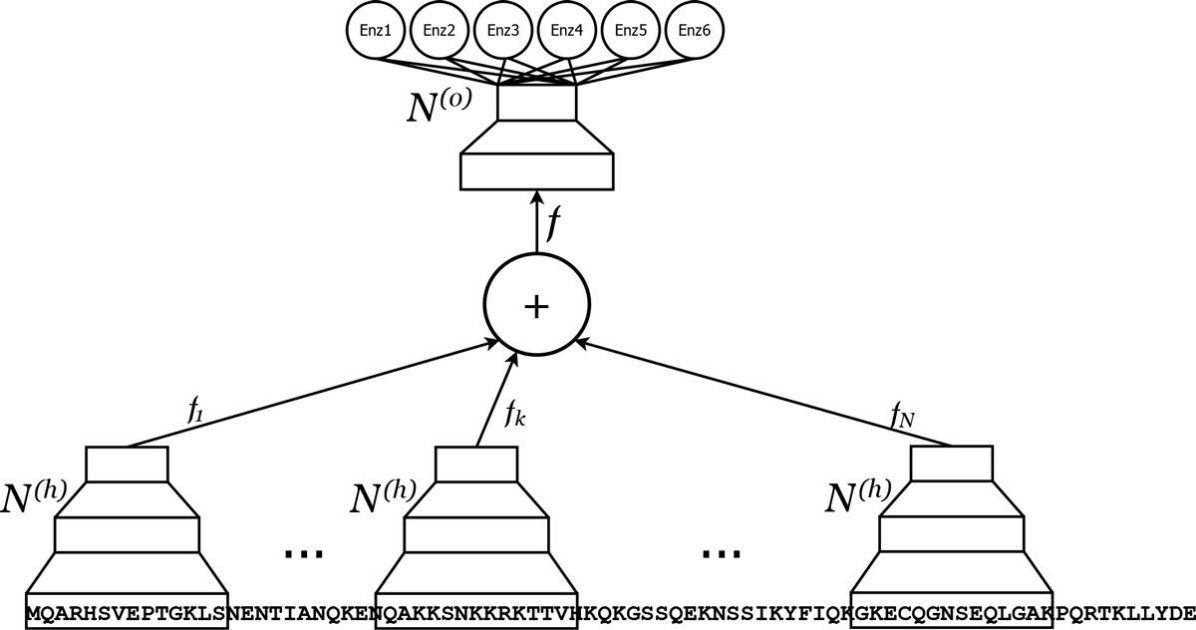
**N-to-1 NN architecture for predicting enzymatic class**. An N-to-1 Neural Network. N copies of the $N^{\left(h\right)}$ network (only 3 represented for simplicity) process all the (overlapping) motifs of a predefined length in a sequence. The vectorial outputs *f_k _*of these networks are added up and the resulting feature vector *f *is input to the $N^{\left(o\right)}$ network to produce the enzymatic class prediction.

The model proposed in this work is a N-to-1 Neural Network based on the previous architecture SCLpred, developed to predict subcellular localization [15], and based on our framework to design Artificial Neural Networks for graphical data [23,24]. The N-to-1 NN model is capable of approximating non-linear functions mapping sequences to features and features to classes in a two-step prediction. In the first step, the sequence-to-feature network is represented by:

$$f=k\overset{N}{\underset{i=1}{\sum }}N^{\left(h\right)}\left(r_{i-c},\ldots ,r_{i+c}\right)$$

where *f *is the feature vector *f *=(*f*_1_, ..., *f_h_*), *r_i _*is the i-th residue in the sequence and $N^{\left(h\right)}$ is a non-linear function implemented by a two-layered feed-forward neural network, with *h *non-linear output units, which takes as input shifting windows of 2*c *+ 1 residues. In all tests in this work we use *c *= 15, corresponding to motifs of 31 residues. Parameter *k *in the equation is a normalizing factor, which we in all tests we set to 10^−2^, although it would be possible in principle to interpret it as a free parameter and fit it to the examples. Thus the network has *I*_1 _= (2 ∗ 15 + 1) ∗ 21 = 651 inputs for the MSA-dataset, and *I*_2 _= (2 ∗ 15 + 1) ∗ 24 = 744 for the MSA + SS-dataset. In this first step the network compresses all 2*c *+ 1 windows of a given sequence into a fixed-size feature vector *f *composed by *h *real-valued descriptors which are automatically learned in order to minimize the output error. The feature-to-output network in the second step takes the feature vector *f *as input and maps it into the property of interest *o *(in this case enzymatic class) according to the following equation:

$$o=N^{\left(o\right)}\left(f\right)$$

where $N^{\left(o\right)}$ is a non-linear function implemented by a second two-layered feed-forward neural network. The whole compound two-step predictive architecture is itself a feed-forward neural network which is trained by gradient descent via the back-propagation algorithm. The free parameters of the overall architecture can be controlled by: $N_{f}^{H}$, the number of hidden units in the first level network; $N_{y}^{H}$, the number of hidden units in the second level network; *N_f_*, the number of hidden states in the feature vector *f*, which is also the number of output units in the sequence-to-feature network. After preliminary experiments, we selected the following structural parameters: $N_{f}^{H}=14$; $N_{y}^{H}=13$; *N_f _*= 12. A graphical representation of an N-to-1 NN is reported in Figure 1.

#### Training

We train our model by 10-fold cross-validation. The dataset is thus split into 10 subsets to perform 10 training runs where a different tenth of the overall set is used as test set. Eight tenths of the overall set are used for training (learning the parameters of the network), one tenth for validation (to monitor the training process) and one tenth for testing (only used for gauging performances, but not to select parameters or hyperparameters of the network). For each fold we create 3 training experiments in which different tenths of the overall set are set aside as validation sets.

Given that some classes are less numerous than others, in each training set we replicate the examples in those classes in order to have approximately the same number of examples in each class.

Training is performed as in [15], by gradient descent on the error modelled as the relative entropy between the target class and the output of the network. The overall output of the network (output layer of *N*^(*o*)^()) is implemented as a softmax function, while all the internal squashing functions are hyperbolic tangents. The examples are shuffled between epochs. The weights in the networks are updated every 10 examples and 1000 epochs of training are performed, which is sufficient to bring the training error down to near zero in all cases. We use a momentum term of 0.9. The learning rate is kept fixed at 0.2 throughout training. For each training, we save the 10 best performing networks (on validation), we ensemble average the total 30 models saved (10 for each of the 3 training versions) and evaluate them on the corresponding test set. The final results for the 10-fold cross-validation are the average of the values obtained on each test set. Training times for each model are approximately 60 hours on a single state of the art core. Without the momentum term we reach similar results, but training times are longer by a factor 4-5, as more training epochs are necessary to reduce the error. Once the system is trained, predictions by the networks are obtained in under one second, two orders of magnitude less than the cost of creating the MSA.

#### Evaluating performance

We evaluate the performance of our predictor using the following measures:

(1) $$\begin{array}{llll} GC & =\sqrt{\frac{\sum_{ij}\frac{{\left(z_{ij}-e_{ij}\right)}^{2}}{e_{ij}}}{N\left(K-1\right)}}\; & & \;\\ Q & =\frac{\sum_{i}z_{ij}}{\sum_{ij}z_{ij}}\; & & \;\\ & \; & \end{array}$$

where:

• *z_ij _*: the number of sequences of class *i *predicted to be in class *j*

• *e_ij _*: the number of sequences of class *i *expected to be predicted in class *j *by chance

• *N *: the number of sequences

• *K *: the number of classes

To calculate the performance for each class we use:

(2) $$\begin{array}{llll} Spec & =\frac{TP}{TP+FP}\; & & \;\\ Sens & =\frac{TP}{TP+FN}\; & & \;\\ FPR & =\frac{FP}{FP+TN}\; & & \;\\ MCC & =\frac{TP\times TN-FP\times FN}{\sqrt{\left(TP+FP\right)\left(TP+FN\right)\left(TN+FP\right)\left(TN+FN\right)}}\; & & \;\\ & \; & \end{array}$$

where:

• True positives (TP): *z_ii_*

• False positives (FP): $\sum_{j\neq i}z_{ji}$

• True negatives (TN): $\sum_{v\neq i}\sum_{j\neq i}z_{jv}$

• False negatives (FN): $\sum_{j\neq i}z_{ij}$

## Results and discussion

In order to examine the overall quality and accuracy of our predictor we average the evaluating measures calculated for each of the 10-fold cross-validation test sets. In Table 2 we compare the performances obtained after training the N-to-1 NN models on the two different input coding schemes used in this work. Our predictor shows a better overall success rate in identifying enzymes among the six main functional classes when trained and tested on the MSA-dataset than on the MSA+SS-dataset. In particular, we observe higher GC values (86% for MSA-dataset and 84% for MSA+SS-dataset) while Q values are 96% for both input encodings. It is not entirely clear why the addition of secondary structure information proves slightly detrimental to the performances of the models. It has been shown that secondary structure does not provide enough information to classify functions [17], and this is especially true for enzymes, which can both exibit large amounts of structural variation within a single class, and, by converse, very different enzymatic activities in spite of nearly identical structures. It is also possible that secondary structure information may be essentially redundant in this case, as it is obtained from the sequence-based predictor Porter [19] and the same sequence is available to the N-to-1 NN. Quick learning based on the secondary structure may bring the hidden units of the model into saturation and hinder further, subtler learning of sequence patterns. This hypothesis is corroborated by faster training times we observe when secondary structure information is included.

**Table 2:** Results after train and test on the two different input coding schemes.

		MSA				MSA+SS		
	**Spec**	**Sens**	**MCC**	**FPR**	**Spec**	**Sens**	**MCC**	**FPR**

Oxidoreductase	0.90	0.89	0.87	0.02	0.88	0.88	0.86	0.02
Transferase	0.90	0.89	0.84	0.05	0.90	0.87	0.82	0.06
Hydrolase	0.89	0.92	0.84	0.07	0.87	0.91	0.82	0.08
Lyase	0.91	0.84	0.87	0.00	0.89	0.82	0.85	0.01
Isomerase	0.81	0.72	0.76	0.01	0.84	0.69	0.76	0.00
Ligase	0.89	0.85	0.86	0.01	0.87	0.84	0.85	0.01

GC	0.86				0.84			

Q	0.96				0.96			

It should be emphasized that the model obtains very high GC values (which weigh all classes equally) and performs accurately even for under-represented classes. Although the best predictions are obtained for the classes with the largest number of examples, such as oxidoreductase (2% FPR and 89% sensitivity), transferase (5% FPR and 89% sensitivity) and hydrolase (7% FPR and 92% sensitivity), the other classes are also well predicted: lyase (0.2% FPR and 84% sensitivity); ligase (1% FPR and 85% sensitivity); and the smallest class isomerase (0.5% FPR and 72% sensitivity).

It has been reported that even for pairs of enzymes with over 70% residue identity in the optimal alignment more than 30% do not belong to the same class (first EC number) [6], underlying the difficulty of obtaining accurate predictions based on sequence identity. However, the overall accuracy of our method in predicting the main enzymatic classes is very high for the datasets used, in which sequence identity is below 30% for any two proteins.

It should also be stated that unlike in profile analysis methods such as [25] we calculate residue frequencies without adding any further information such as weights derived from the mutational distance matrix. In future research we will be investigating whether richer input coding may improve our predictions further. Moreover, contrary to feature-based methods such as [13], we do not apply any explicit feature selection process. N-to-1 NN implicitly operate feature selection on the sequence, as information from all different motifs in a protein effectively "compete" to be represented into a small feature vector. Whether this fixed-sized representation of proteins may shed light on the functional space of proteins, and whether fully trained networks may be analyzed to extract functional motifs on which predictions are based, are two future direction of research we are currently pursuing.

We qualitatively compare our results with the performance of the ProtFun method [13] that makes use of Neural Networks as predictive architecture to combine prefixed features from Human protein sequences and to correlate them to six enzymatic classes defined according to EC classification scheme. Both predictors are assessed by 10-fold cross-validation and adopt the same class definition, but our neural network is trained and tested on a larger dataset than the one used to train ProtFun. Although tests on different datasets should always be taken with caution, overall our method is far more accurate than ProtFun and shows a more balanced sensitivity and a considerably lower FPR across classes. According to what it is reported for ProtFun's results in [13], at a level of thresholding giving 70% correct predictions, the range of false positives varies from below 10% to below 40%, with most classes giving about 20% false positives. Our predictor, instead, achieves better performances showing correct predictions over 80% and a range of false positives below 10% for five of the six classes, and a correct prediction around 70% with a fraction of false positives below 10% for the smallest class (isomerase). A class-by-class comparison of our predictor and ProtFun is reported in Table 3. Results for ProtFun are derived from a graph in the original paper and are, as such, approximate.

**Table 3:** Results for MSA and ProtFun (from [13]) trained and tested in 10-fold cross-validation.

	MSA		ProtFun	
	**Sens**	**FPR**	**Sens**	**FPR**

Oxidoreductase	0.89	0.02	0.62	0.25
Transferase	0.89	0.05	0.65	0.20
Hydrolase	0.92	0.07	0.60	0.20
Lyase	0.84	0.00	0.71	0.15
Isomerase	0.72	0.01	0.75	0.17
Ligase	0.85	0.01	0.87	0.10

## Conclusions

One of the most important challenges of Bioinformatics at present and for the foreseeable future is to develop accurate computational methods capable of annotating the massive amount of proteins with unknown function deriving from complete sequenced genomes. When dealing with lack of significant sequence homology between two proteins, it is hard to transfer functional annotations reliably. Moreover, divergent/convergent evolutionary events make this task more difficult [26]. Among function prediction tools, sequence frequency profile methods are the most effective at detecting evolutionarily and functionally significant residue patterns (e.g. active-site residues/portion) which can be properly harnessed for enzymatic class prediction.

In this article, we presented a predictive method based on a novel Neural Network architecture able to map a sequence into a vector of properties and to extract relevant information from amino acid frequencies derived from multiple sequence alignments. We have developed one kingdom specific predictor for animals into six classes for enzymatic sequences. We have trained and tested our method in 10-fold cross-validation on large non-redundant subsets of annotated enzymes from UniprotKB/SwissProt according to the EC classification scheme.

Encoding inputs correctly and effectively is conductive to improved performances. In our case we are capable of extracting information both from residue frequencies at a given sequence site and from long patterns of residues which are considered without loss of positional information. Although we do not know yet which patterns are stored while automatically compressing the sequence into a feature vector, and we will investigate the matter in the future, the high classification performances we achieve suggest that the network is able to recognize those functionally conserved portions of enzymatic sequences that are related to the reaction on which the EC classification scheme is based. We compared our method to ProtFun which has been shown to be one of the most accurate methods for function prediction and, although comparisons on different datasets are to be taken with caution, we obtained performances exceeding those of ProtFun by over 10% while also considerably reducing false positive rates. We also investigated a richer input encoding which includes predicted secondary structures as an additional input. In this case we did not observe an increase of the overall accuracy, probably due to lack of relevant additional information. We intend to investigate other sources of additional information (e.g. solvent accessibility, intrinsic disorder, contact density [27,28], location of binding sites, post-translational modifications) in our future research. Although we have obtained state of the art performances, we aim to extend the training on a wider dataset. We expect this to be a beneficial to our network because, unlike other models that work solely on simpler features such as single-residue frequencies, it has a higher number of free parameters and the ability to detect complex, long patterns of residues. We are currently working on a extensive dataset that comprises every EC sequence of the ENZYME database without kingdom distinction and a further direction is to solve the challenging aspect of predicting the second and the third levels of EC classification.

## Competing interests

The authors declare that there are no competing interests.

## Authors' contributions

VV has designed the datasets, trained and tested the models and written the first draft of this manuscript.

AA has contributed to the code, to the design of the experiments and to the revision of the manuscript.

GP has designed and implemented the original code for N-to-1 NN, has directed the work and has produced the final version of the manuscript. All authors approve the contents of this article.

## Declarations

The publication costs for this article were funded by University College Dublin through a UCD Seed Funding 2011 award to GP.

This article has been published as part of *BMC Bioinformatics *Volume 14 Supplement 1, 2013: Computational Intelligence in Bioinformatics and Biostatistics: new trends from the CIBB conference series. The full contents of the supplement are available online at http://www.biomedcentral.com/bmcbioinformatics/supplements/14/S1.
